# Impact of Dietary Salicylates on Iron, Zinc, and Copper Status in Preeclampsia Model Rats Induced by L-NAME

**DOI:** 10.1007/s12011-025-04772-1

**Published:** 2025-08-07

**Authors:** Rafsan Syabani Cholik, Katarzyna Skrypnik, Agnieszka Waśkiewicz, Marta Karaźniewicz-Łada, Joanna Suliburska

**Affiliations:** 1 Department of Human Nutrition and Dietetics, Poznan University of Life Sciences, Poznań, Poland; 2 Department of Chemistry, Poznan University of Life Sciences, Poznań, Poland; 3 Department of Physical Pharmacy and Pharmacokinetics, Poznan University of Medical Science, Poznań, Poland

**Keywords:** Dietary salicylates, Iron, Zinc, Copper, Preeclampsia, L-NAME

## Abstract

Low-dose aspirin prophylaxis is recommended for women at high risk of preeclampsia. It has been suggested that dietary salicylates may have a similar effect. Despite the known anti-inflammatory properties of salicylates, their influence on trace elements in preeclampsia remains unclear. This research investigated the effect of dietary salicylates and aspirin on iron, zinc, and copper status in rats with NG-nitro-L-arginine methyl ester (L-NAME)–induced preeclampsia. The study involved pregnant Sprague Dawley rats divided into six groups: control group (CH), preeclamptic rats (CP), preeclamptic rats with a low dose of dietary salicylate (LSP), preeclamptic rats with a high dose of dietary salicylate, preeclamptic rats with a low dose of aspirin (LAP), and preeclamptic rats with a high dose of aspirin. The content of trace elements in diets, liver, kidney, heart, spleen, pancreas, femur, brain, and hair was measured using flame atomic absorption spectrometry. Salicylate concentrations in diets, serum, and urine were analyzed using HPLC and UHPLC-MS/MS systems. Administration of L-NAME resulted in elevated blood pressure across groups, and only the LAP group had blood pressure levels comparable to the CH group. Preeclampsia significantly decreased serum hepcidin levels, while salicylates abolished this effect. Salicylate administration significantly decreased iron levels in hair and increased maternal zinc concentrations in the brain. Dietary salicylates markedly increased zinc levels in the placenta. In conclusion, L-NAME–induced preeclampsia decreases maternal serum hepcidin. Treatment with salicylates modulates iron and zinc status in preeclamptic rats, with specific effects on hepcidin levels.

## Introduction

Preeclampsia is a complex hypertensive syndrome that develops after 20 weeks of gestation and is characterized by high systemic blood pressure, proteinuria, and organ dysfunction [1]. Inflammation and oxidative stress are key pathophysiological mechanisms in preeclampsia and significantly influence the regulation and utilization of micronutrients, which serve as essential cofactors for antioxidant enzymes and participate in various cellular metabolic pathways [2, 3]. Additionally, preeclampsia is a complex medical syndrome for which curative and preventive strategies remain inconclusive. Treatment is largely symptomatic and includes antihypertensive drugs, magnesium sulfate to prevent seizures, and prompt delivery, depending on gestational age and disorder severity [4, 5]. It is currently suggested that low-dose aspirin may reduce the risk of developing preeclampsia [4, 6–10].

Due to their structural similarity to aspirin, dietary salicylates are thought to have comparable protective properties [7]. To better understand their potential roles in regulating platelet aggregation, inflammation, and oxidative stress—particularly about mineral homeostasis and the pathophysiology of preeclampsia—further studies are needed [11]. Dietary salicylates are naturally occurring plant phenolic compounds found widely in fruits, vegetables, spices, and herbs. They share structural similarities with acetylsalicylic acid and possess antithrombotic and anti-inflammatory properties [12].

Several studies have shown that mineral balance is important in the prevention of preeclampsia. Some authors have reported elevated copper, iron, and ferritin levels in serum and urine, along with reduced zinc levels, in preeclamptic women compared to healthy pregnant women [13, 14]. Iron (Fe), zinc (Zn), and copper (Cu) are key regulators of vascular function and act as cofactors in antioxidant defense mechanisms [2, 15, 16]. Animal model experiments have confirmed that misregulation of these essential micronutrients is associated with oxidative injury, endothelial dysfunction, and placental growth inhibition—particularly in conditions involving nitric oxide synthase inhibitors, such as L-NAME. These features are commonly observed in the clinical syndrome of preeclampsia [17].

The pro-oxidant effect of iron, due to its role in the Fenton reaction and the subsequent production of ROS, may lead to placental and vascular tissue damage, supporting the reported associations between high serum or hepatic iron and increased lipid peroxidation [18]. Additionally, according to Olechnowicz et al., low zinc levels have been shown to impair the activity of antioxidant enzymes such as superoxide dismutase (SOD), thereby exacerbating oxidative stress and inflammatory responses [19]. Experimental studies have demonstrated that zinc supplementation can reduce preterm birth and restore antioxidant function, highlighting its protective potential [20]. In contrast, elevated copper levels may exert pro-oxidant effects, particularly in the context of zinc deficiency [21].

It is important to note that both aspirin and dietary salicylates can interfere with micronutrient absorption, transport, and gastrointestinal utilization, emphasizing the need for further research into their effects on iron, zinc, and copper homeostasis [22, 23]. However, the mechanisms by which salicylates influence trace-element balance in preeclampsia remain poorly understood.

Presumably, dietary salicylates may exert aspirin-like effects in preventing preeclampsia by enhancing placental blood flow and reducing inflammation [24, 25]. According to the literature, iron, zinc, and copper may play significant roles in the development of preeclampsia [26–29]. Therefore, this study aims to evaluate iron, zinc, and copper status in an animal model of preeclampsia and to assess whether dietary salicylates and aspirin influence trace element status in preeclamptic rats.

## Material and Methods

### Animals

A total of 48 female and 20 male Sprague Dawley rats were obtained from Charles River Laboratories, Germany. Female rats were 12 weeks old, with a mean weight of 288 ± 25.52 g. The experiment followed the National Institutes of Health *Guide for the Care and Use of Laboratory Animals* (National Institutes of Health Publications No. 80–23, Revised 1978), the European Communities Council Directive of 24 November 1986, and Polish legal regulations. The procedures were approved by the Local Bioethics Committee (approval no. 6/2023). The ARRIVE guidelines (Animal Research: Reporting of In Vivo Experiments) were followed. The animals were acclimated to the laboratory environment for 10 days. During this period, they had unrestricted access to deionized water and AIN-93 M food (Zoolab, Sędziszów, Poland). The rats were maintained under controlled settings. The ambient temperature was regulated at 21 ± 1 °C with humidity levels between 55 and 65%, and the light/dark cycles were 12 h each. The animals were kept individually in stainless steel cages.

### Experimental Design

The experiment was performed on pregnant rats. To facilitate copulation, a female rat in estrus was confined with male rats, and pregnancy was confirmed by the presence of a mucous plug in the vagina (gestational day 0, GD0). Pregnant rats were randomly assigned to the following six groups (*n* = 8 per group):

- CH: a control group of pregnant rats
- PE: pregnant rats administered L-NAME (NG-nitro-L-arginine methyl ester)
- LSP: pregnant rats administered L-NAME and a low dose of dietary salicylates
- HSP: pregnant rats administered L-NAME and a high dose of dietary salicylates
- LAP: pregnant rats administered L-NAME and a low dose of aspirin
- HAP: pregnant rats administered L-NAME and a high dose of aspirin

Preeclampsia was induced by administering deionized water containing 0.5 mg/mL L-NAME (N5751, Merck, Sigma-Aldrich, Darmstadt, Germany) from gestational day 6 to gestational day 19 [30]. Dietary salicylates and aspirin were incorporated into the feed. A mixture of products with a relatively high salicylate content was added to the AIN-93G diet (Zoolab, Sędziszów, Poland) to create low- and high-salicylate diets. The mixture consisted of 50% buckwheat groats, 10% oregano, 10% basil, 10% cumin, 10% tarragon, and 10% mint leaves, all sourced commercially in Poland [12].

For the LSP group, 1% of the mixture was added to the diet, and for the HSP group, 10% was added. Aspirin doses were determined by analyzing the salicylate content of the LSP and HSP diets, ensuring comparability between dietary salicylates and aspirin. Commercially available aspirin (Aspirin®, Bayer) was used.

**Fig. 1:**
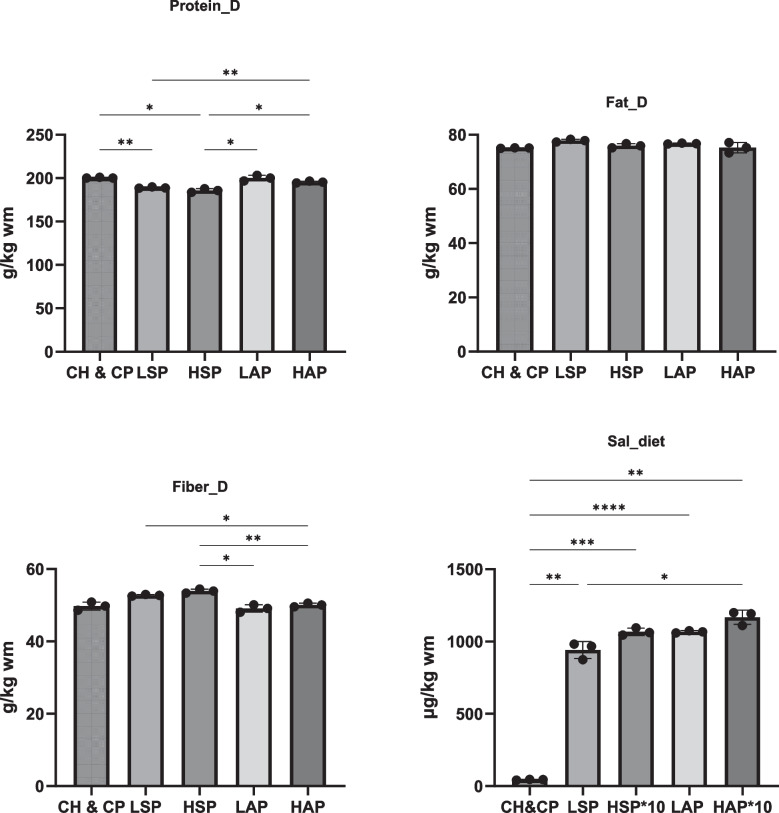
Diet composition. D, diet; *p < 0.05; **p < 0.01; ***p < 0.001

**Fig. 2:**
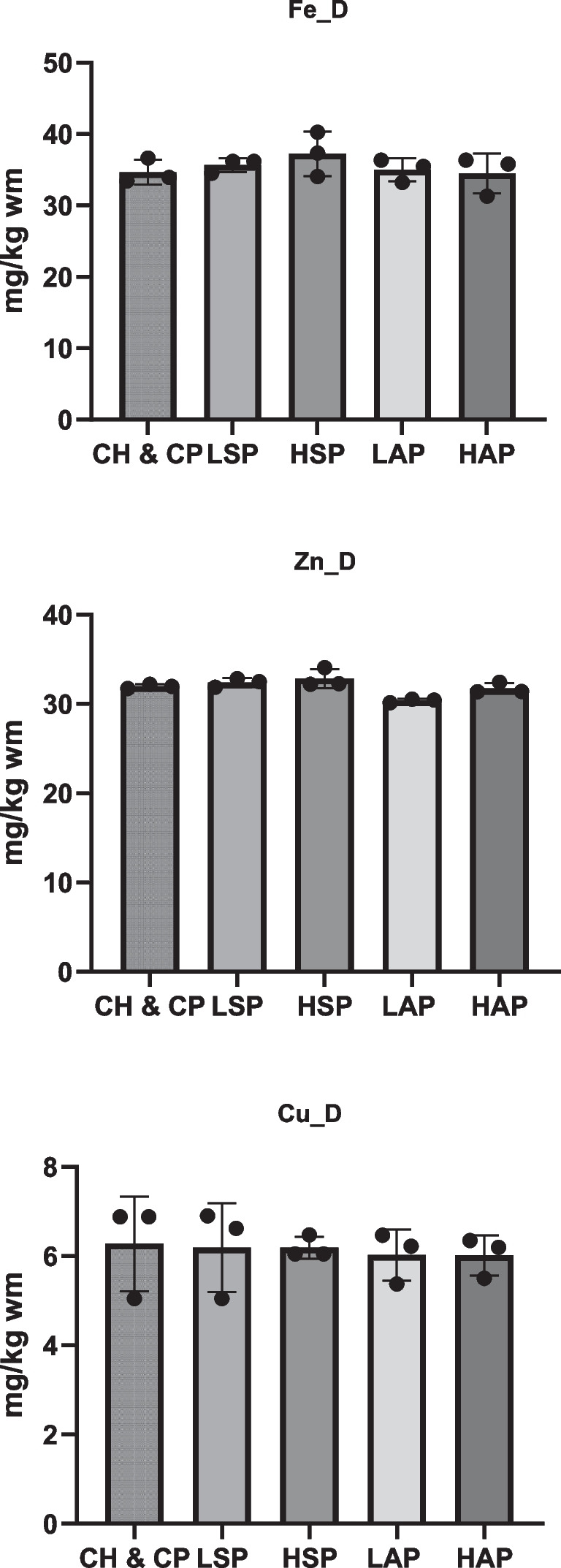
Mineral content in diets. D, diet

The experimental period coincided with the rat gestation period, lasting from GD0 to GD19. During this duration, rats had unrestricted access to deionized water and their assigned diet. Food intake was monitored daily, and body weight was recorded weekly. The complete composition of the diets and total salicylate content is provided in Figs. 1 and 2. Rats were weighed and decapitated after the experimental period (GD19).

### Diet Composition

Diet samples were analyzed in triplicate according to AOAC (Association of Official Analytical Chemists) methods. Protein content was determined using a Kjel-Foss Automatic 16210 analyzer (Foss Electric, Hillerød, Denmark). Fat content (ether extract, EE) was measured with a Soxhlet System HT analyzer (Foss Electric, method no. 973.18). The fiber was analyzed using amylase and sodium sulfite, expressed without residual ash, with the Fibertech 1020 Analyzer (Foss Analytical AB, Höganäs, Sweden) [31].

### Salicylates in Diets

Total salicylates were extracted from 0.5 g of homogenized dietary samples using methanol for 20 min with vortexing and sonication. Following centrifugation, the methanol extracts were split equally to assess free salicylic acid (SA) and its glucoside (SAG). The solvent was evaporated under a stream of nitrogen, and the residue was dissolved in 5% trichloroacetic acid. After further centrifugation, SAG was extracted three times into an organic phase consisting of ethyl acetate, cyclopentane, and isopropanol (100:99:1, v/v/v). For the determination of total salicylate content, SAG was enzymatically degraded using 40 units of β-glucosidase in acetate buffer (0.1 M, pH 5.2).

After evaporation of the solvent, the dry residue was dissolved in a mobile phase consisting of 0.2 M acetate buffer with 0.5 mM EDTA (v/v, pH 5.0), filtered through a 0.20 µm syringe filter (Chromafil, Macherey–Nagel, Düren, Germany) and analyzed by LC. SA was quantified using a Waters LC system (Waters, Manchester, MA, USA) equipped with a 2475 Multi-λ fluorescence detector and a Waters HPLC Symmetry C18 column (Waters, Manchester, MA, USA). The mobile phase flow rate was 0.8 mL/min, and the retention time was 8.2 min.

### Blood Pressure Measurement

Blood pressure measurements were conducted on gestational days 6 (GD6) and 18 (GD18) using a non-invasive blood pressure monitoring system (CODA-PCS42, Kent Scientific, USA). Blood pressure was assessed via the tails of the rats using Volume Pressure Recording (VPR) sensor technology. To enhance tail blood circulation, rats were placed on heating blankets at 38 °C for 15 min. For each animal, the mean blood pressure was calculated from an average of seven recorded readings. The rats were acclimated to the measurement device and exhibited no distress during the inflation–deflation cycles.

### Sample Collection

Urine samples were collected on GD6 and GD18 at a consistent time in the morning, while the rats had unrestricted access to food. Approximately 0.5–0.7 mL of urine was collected from each rat. Glass pads and a pipette were used to collect the urine. The samples were stored at − 80 °C. Blood samples in the amount of approximately 2 mL were collected in serum-separating tubes and left at room temperature for 30 min before centrifugation at 2000 rpm for 15 min at 4 °C. The resulting supernatant was collected and stored at − 80 °C. Placentas, fetuses, liver, kidneys, heart, spleen, pancreas, femur, and brain were dissected, weighed, and stored at − 80 °C. Hair was collected from the same anatomical area (the interscapular region) of each rat. Hair was cut with steel-free scissors, and approximately 400–500 mg of hair was collected from each rat.

### Salicylates in Serum and Urine

Concentrations of total salicylates were measured using a validated UHPLC-MS/MS method. Serum samples were prepared by mixing with formic acid (FA) solution in methanol (2%) and water (12%), along with an internal standard solution. Liquid–liquid extraction was carried out using ethyl acetate. The mixture was shaken and centrifuged, after which the organic phase was evaporated in a vacuum concentrator (Eppendorf, Germany). The resulting dry residue was dissolved in 2% FA solution in water.

For urine sample preparation, urine was mixed with methanol and an internal standard solution at a concentration of 1 µg/mL. The samples were vortex-mixed and centrifuged, and an aliquot of the supernatant was diluted with 2% FA in water.

An aliquot of each prepared sample was injected into a Nexera UHPLC system coupled to an LCMS-8030 Triple Quadrupole tandem mass spectrometer (Shimadzu, Japan). Analytes were separated on a Luna 3 µm Phenyl-Hexyl 100 Å LC column (Phenomenex, USA) at 25 °C. The mobile phase consisted of deionized water (A) and acetonitrile (B), both containing 0.1% FA. The gradient program was as follows: 0–5 min, linear increase from 20 to 80% B; 5–6 min, 80% B; 6–7 min, decrease to 20% B; post-time, 1 min at 20% B for column equilibration.

The mobile phase flow rate was 0.4 mL/min. MS detection was performed using electrospray ionization: positive ion mode for GA and negative ion mode for SA, SA-d4, and SCA. Detector parameters were as follows: desolvation line temperature, 235 °C; heat block temperature, 400 °C; nebulizing gas flow, 2 L/min; drying gas flow, 10 L/min; and electrospray needle voltage, 4.5 kV.

### Urinary Albumin

Albumin content in urine was measured using commercial enzyme-linked immunosorbent assay (ELISA) kits (Qayee Bio-Technology Co., Ltd., Shanghai, China). Spectrometry was performed using an Infinite F50 spectrometer (Tecan Group Ltd., Männedorf, Switzerland).

Measurement precision was verified by analyzing two standards and comparing their calculated concentrations to nominal values. Additionally, three random rat samples were analyzed in duplicate, and their measured values were compared. In all procedures, the coefficient of variation was below 5%. A control sample provided by the kit manufacturer was used to verify reproducibility.

### Iron, Zinc, and Copper in Diet, Tissues, and Hair

The iron, zinc, and copper content in the diets and tissues was determined using flame atomic absorption spectrometry (Atomic Absorption Spectrophotometer ZA3000, Hitachi, Tokyo, Japan) after digestion in 65% (w/w) spectrally pure HNO_3_ (Merck, Kenilworth, NJ, USA) using a microwave digestion system (Mars 2™ System; CEM Corporation, USA), followed by dilution with deionized water.

Hair samples were washed with acetone and deionized water with subsequent drying on air to a stable weight. The hair samples were then mineralized in a microwave oven as described above.

Trace element concentrations were measured at the following wavelengths: 248.3 nm for iron, 213.9 nm for zinc, and 324.8 nm for copper. The accuracy of the method was verified using certified reference materials: Brown Bread BCR191 (LGC Standards GmbH, Wesel, Germany) for diet samples and bovine liver—trace elements, NIST-1577C, CERT (Sigma-Aldrich, Saint Louis, MO, USA) for tissue samples. Method accuracies were 95–97% for iron, 92–95% for zinc, and 99–103% for copper.

### Biochemical Parameters in Serum and Placenta

Serum and placental concentrations of ceruloplasmin, metalloproteinase 9 (MMP9), superoxide dismutase (SOD), ferritin, and hepcidin were measured using commercial enzyme-linked immunosorbent assay (ELISA) kits (Qayee Bio-Technology Co., Ltd., Shanghai, China). Spectrometry was performed using an Infinite F50 spectrometer (Tecan Group Ltd., Männedorf, Switzerland).

Measurement accuracy was assessed by analyzing two standards and comparing the calculated concentrations with nominal values. Moreover, three randomly selected rat samples were assayed in triplicate, and the results were compared. In all procedures, the coefficient of variation was less than 5%. Reproducibility was verified using a control sample supplied by the kit manufacturer.

Placental samples were homogenized using an automatic homogenizer (MagNA Lyser, Roche, Basel, Switzerland). Biochemical parameters were quantified in the resulting homogenate.

### Statistical Analysis

Data analysis was performed using GraphPad Prism version 10.4.2 (Boston, MA, USA). Results are presented in figures as means with standard deviations. The Shapiro–Wilk test was used to assess normality. For normally distributed data, one-way ANOVA with Tukey’s post hoc test was applied; for non-normally distributed data, the Kruskal–Wallis test with rank sum comparisons was used. Statistical significance was set at *p* < 0.05.

Correlation analysis was performed using Pearson’s method for normally distributed parameters and Spearman’s method for non-normally distributed parameters. A sample size of eight rats per group was calculated to provide 90% power to detect statistical significance at an *α* level of 0.05.

## Results

The composition of the diets is presented in Figs. 1 and 2. Fat content was comparable across all groups (Fig. 1), while protein levels were lower in the modified diets than in the standard diet. The diet with a high amount of dietary salicylates (HSP) contained the lowest protein level and the highest fiber content. The total salicylate content in the diets for the LSP and LAP groups was approximately 1 mg/kg, while the content in the HSP and HAP groups was ten times higher. Iron, zinc, and copper content in the diets did not differ significantly between groups (Fig. 2).

**Fig. 3:**
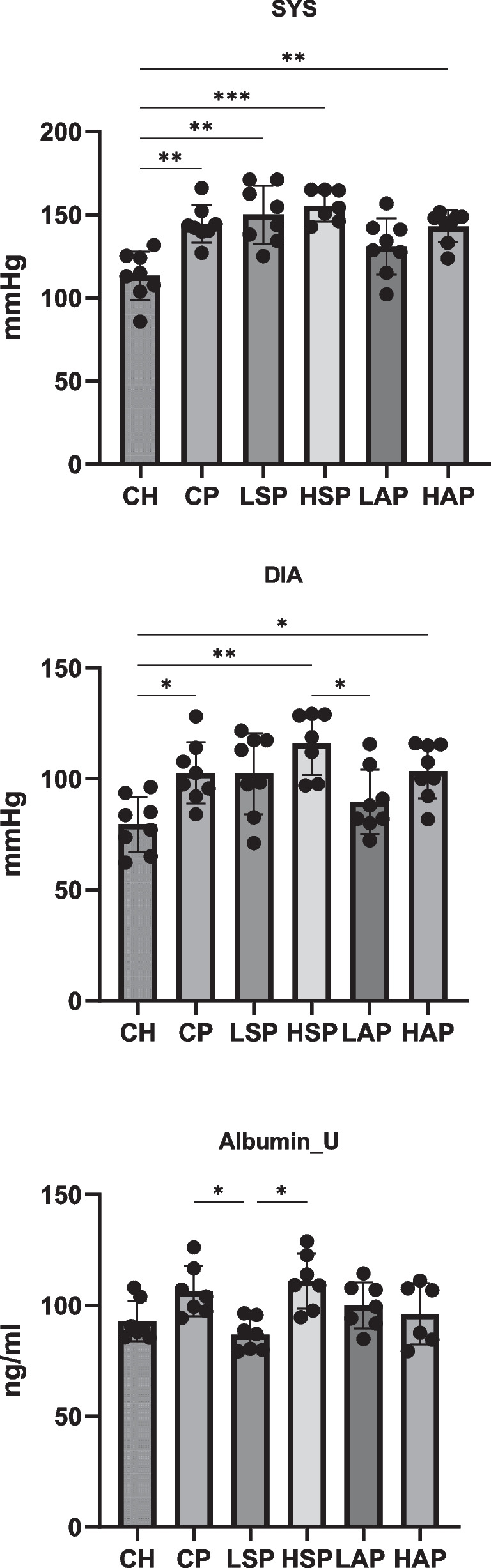
Blood pressure and album concentration in urine. SYS, systolic blood pressure; DIA, diastolic blood pressure; U, urine; *p < 0.05; **p < 0.01; ***p < 0.001

**Fig. 4:**
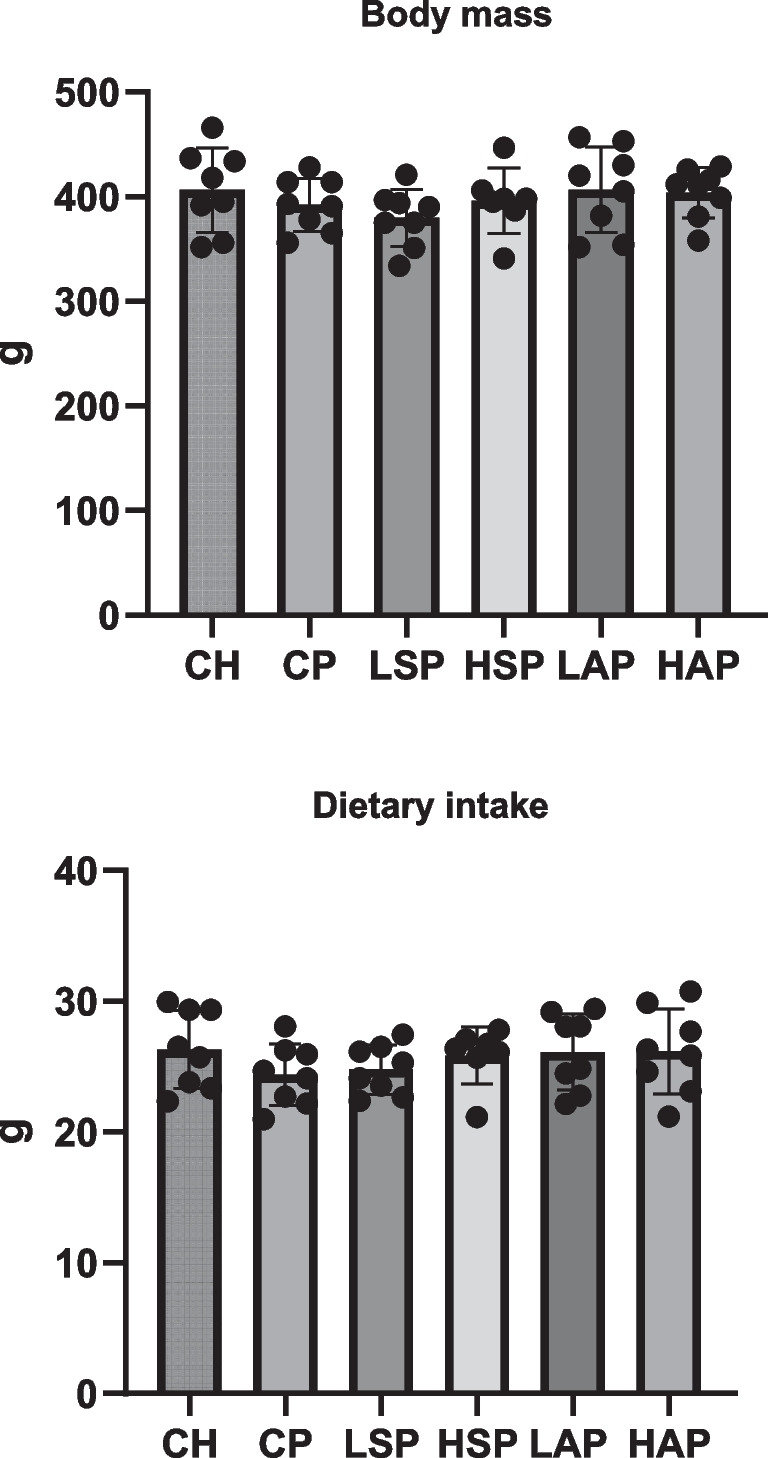
Body mass and dietary intake

Treatment with L-NAME significantly increased blood pressure in rats, but only the administration of low-dose aspirin reduced SYS and DIA pressure to levels comparable to the control group (Fig. 3). A slight increase in urinary albumin concentration was observed in rats with induced preeclampsia. Significantly lower urinary albumin concentrations were found in the LSP group compared to the CP and HSP groups (Fig. 3). Average dietary intake and final body weight did not differ significantly across groups (Fig. 4).

**Fig. 5:**
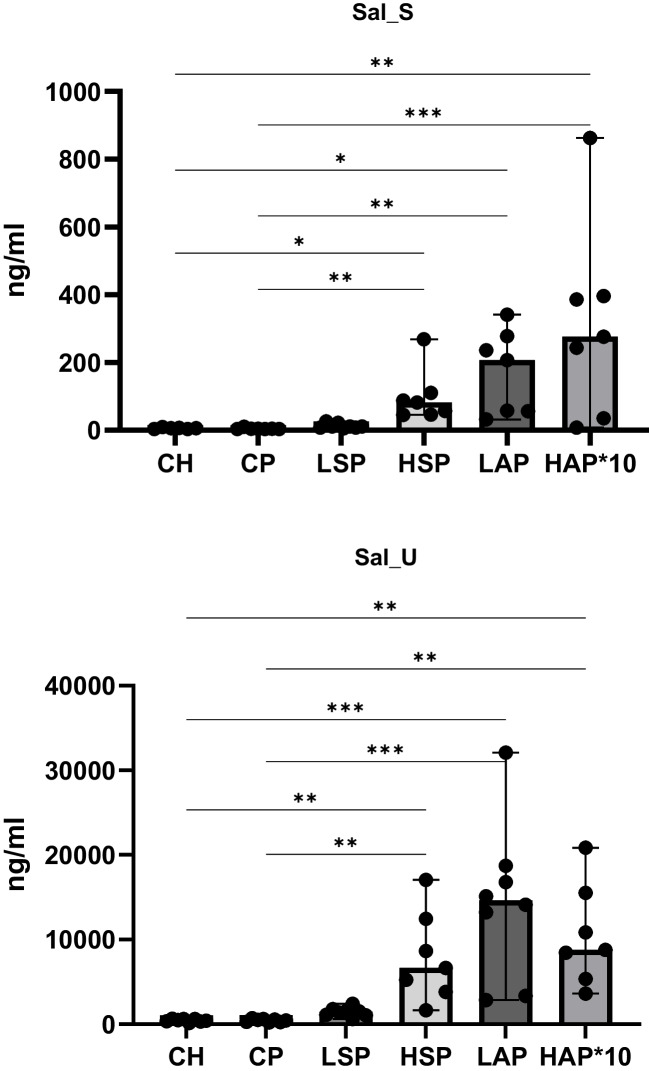
Salicylate concentration in serum and urine. Sal, salicylates; S, serum; U, urine; *p < 0.05; **p < 0.01; ***p < 0.001

Serum and urine concentrations of total salicylates are presented in Fig. 5. Significantly higher salicylate levels were observed in the HAP, HSP, and LAP groups compared to the CH, CP, and LSP groups. The highest salicylate concentrations in serum and urine were noted in the LAP and HAP groups, while levels in the LSP group were comparable to those in the CH and CP groups.

**Fig. 6:**
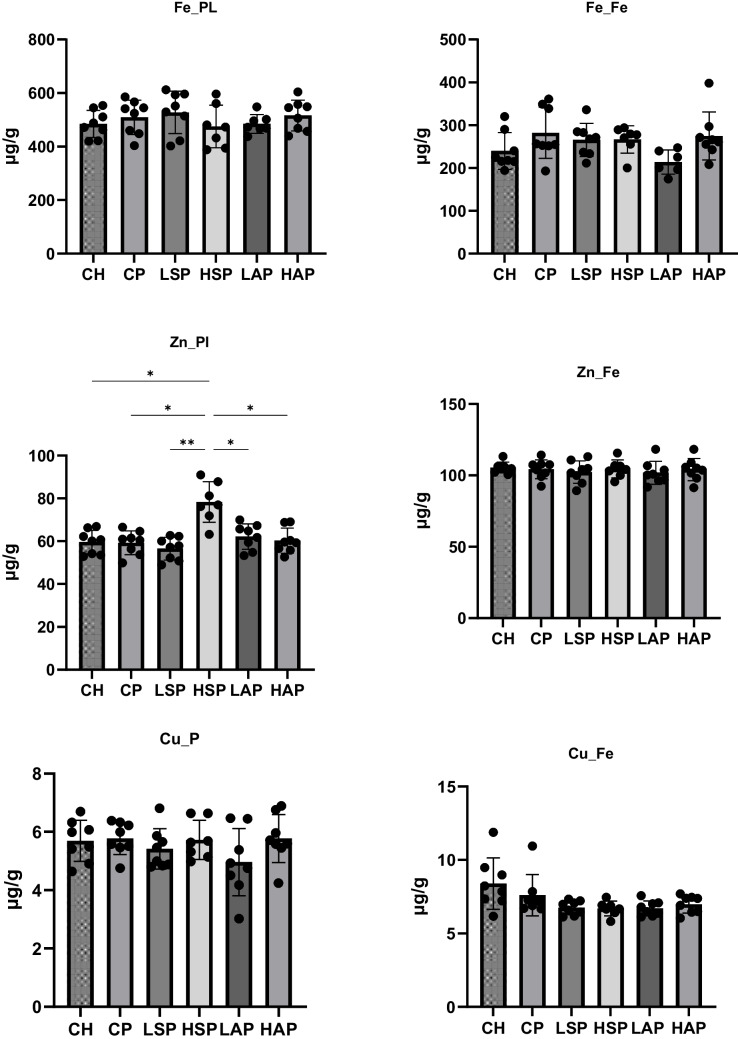
Concentration of minerals in placenta and fetus. Pl, placenta; Fe, fetus; *p < 0.05; **p < 0.01

**Fig. 7:**
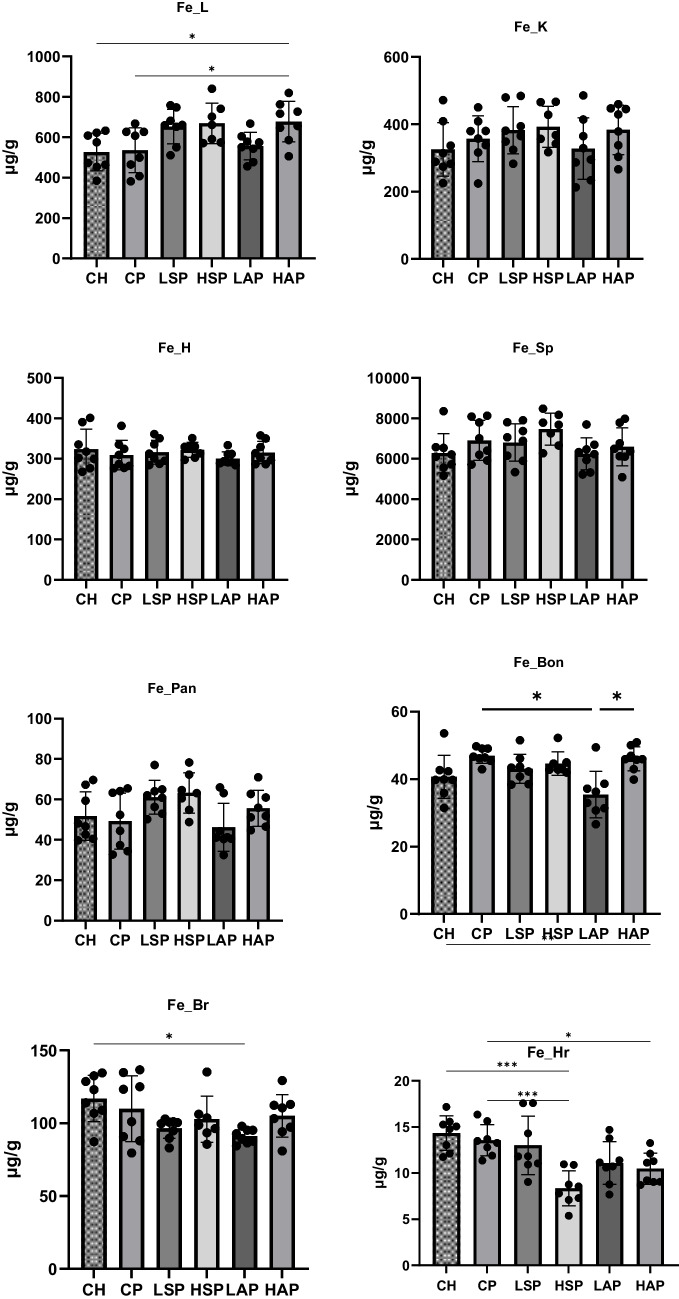
Iron content in tissues. L, liver; K, kidney; H, heart; Sp, spleen; Pan, pancreas; Bon, femur; Br, brain; Hr, hair; *p < 0.05; ***p < 0.001

Administration of L-NAME did not significantly affect iron content in maternal and fetal tissues (Fig. 6). However, a trend was observed in salicylate-treated groups, with increased liver iron and decreased hair iron content (Fig. 7). The lowest iron levels were tendentially observed in the LAP group. Significant differences in tissue iron concentrations were noted between groups. In the HAP group, liver iron content was significantly higher than in the CH and CP groups. In the LAP group, brain iron concentration was significantly lower than in the CH group, while bone iron content was significantly lower than in the CP and HAP groups. High doses of salicylates (HAP and HSP groups) significantly reduced hair iron levels compared to the CH and CP groups.

**Fig. 8:**
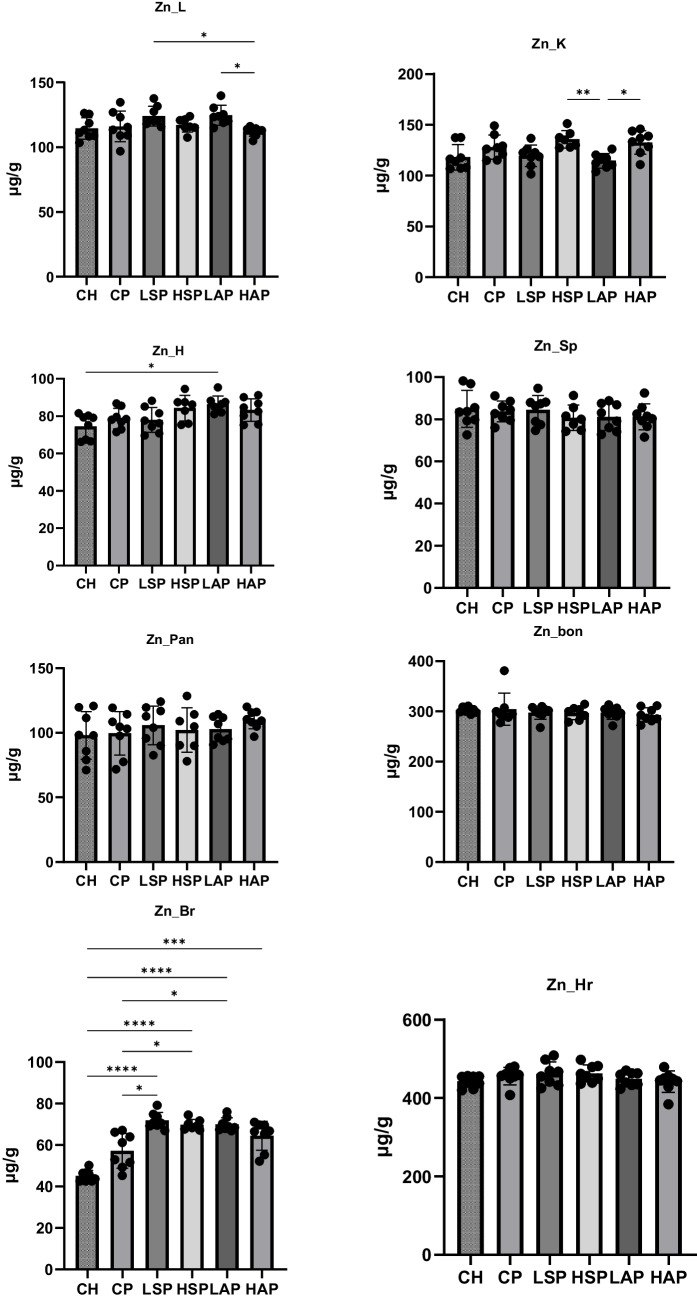
Zinc content in tissues. L, liver; K, kidney; H, heart; Sp, spleen; Pan, pancreas; Bon, femur; Br, brain; Hr, hair; *p < 0.05; **p < 0.01; ****p < 0.0001

In the case of zinc, preeclampsia had no significant effect on tissue concentrations. However, salicylates significantly increased zinc concentrations in the brain compared to standard diet groups (Fig. 8). Higher salicylate content in the diet also significantly increased placental zinc levels (Fig. 6), with no change in fetal zinc content. The lowest liver zinc concentration was found in the HAP group and was significantly lower than in the LAP and LSP groups. In contrast, the kidney zinc level was lowest in the LAP group and significantly differed from the HSP and HAP groups. Zinc levels in the heart were markedly higher in the LAP group than in the CH group (Fig. 8).

**Fig. 9:**
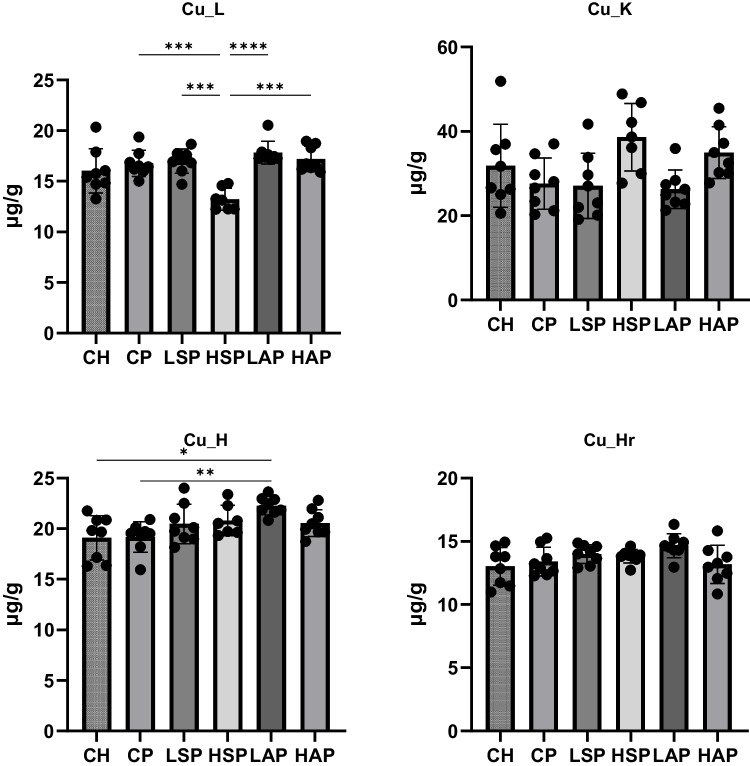
Copper content in tissues. L, liver; K, kidney; H, heart; Sp, spleen; Pan, pancreas; Bon, femur; Br, brain; Hr, hair; *p < 0.05; **p < 0.01; ****p < 0.0001

The level of copper was assessed only in selected tissues, as concentrations in the spleen, femur, brain, and pancreas were below the limit of detection (< LOD). Among the L-NAME–treated groups, the copper content was significantly lowest in the HSP group (Fig. 9). In contrast, markedly higher copper content was observed in the heart of the LAP group compared to the CP and CH groups (Fig. 9).

**Fig. 10:**
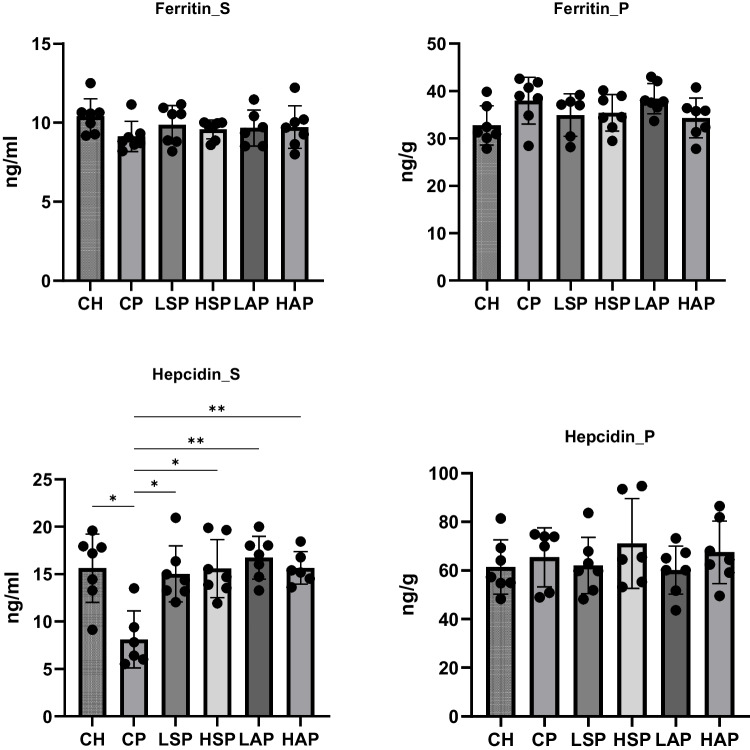
Ferritin and hepcidin level in serum and placenta. S, serum; P, placenta

**Fig. 11:**
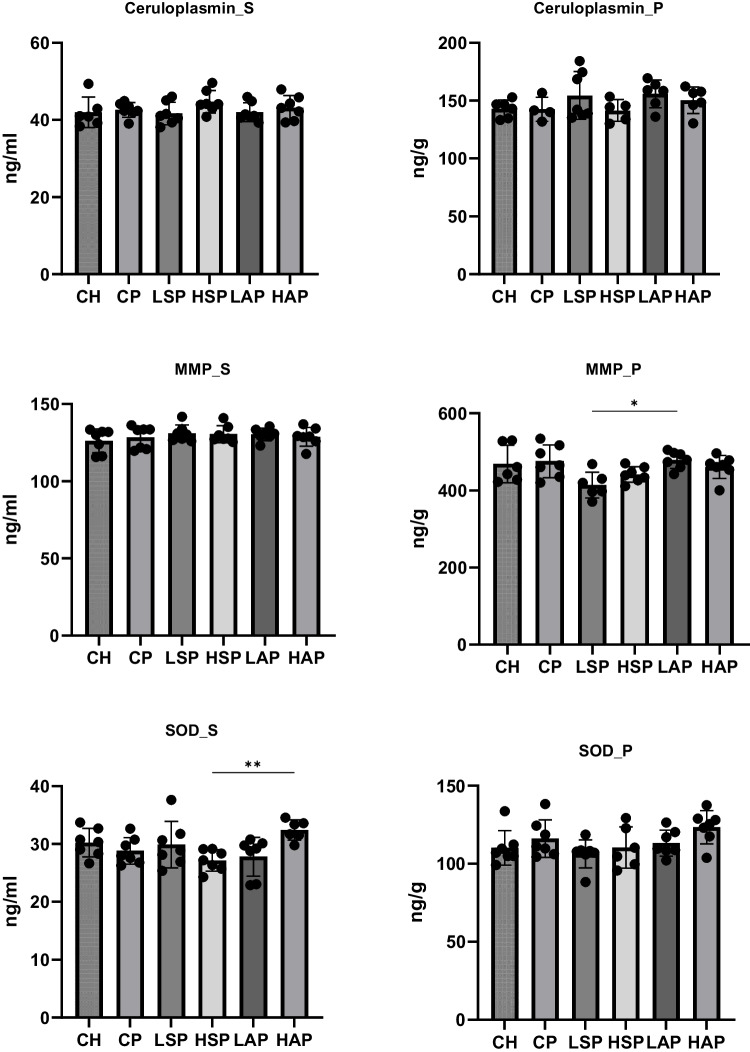
Ceruloplasmin, MMP-9, and SOD concentration in serum and placenta. S, serum; P, placenta; MMP, metalloproteinase 9; SOD, superoxide dismutase; *p < 0.05; **p < 0.01

The study also analyzed biochemical parameters physiologically associated with the measured elements, including ceruloplasmin, MMP-9, SOD, ferritin, and hepcidin (Figs. 10 and 11). The most notable change was observed in serum hepcidin concentration. Preeclampsia significantly reduced serum hepcidin levels, while diet-modified groups maintained hepcidin concentrations comparable to the CH group (Fig. 11). Moreover, the serum SOD concentration was significantly higher in the HAP group than in the HSP group. MMP-9 content in the placenta was significantly higher in the LAP group than in the LSP group.

**Fig. 12:**
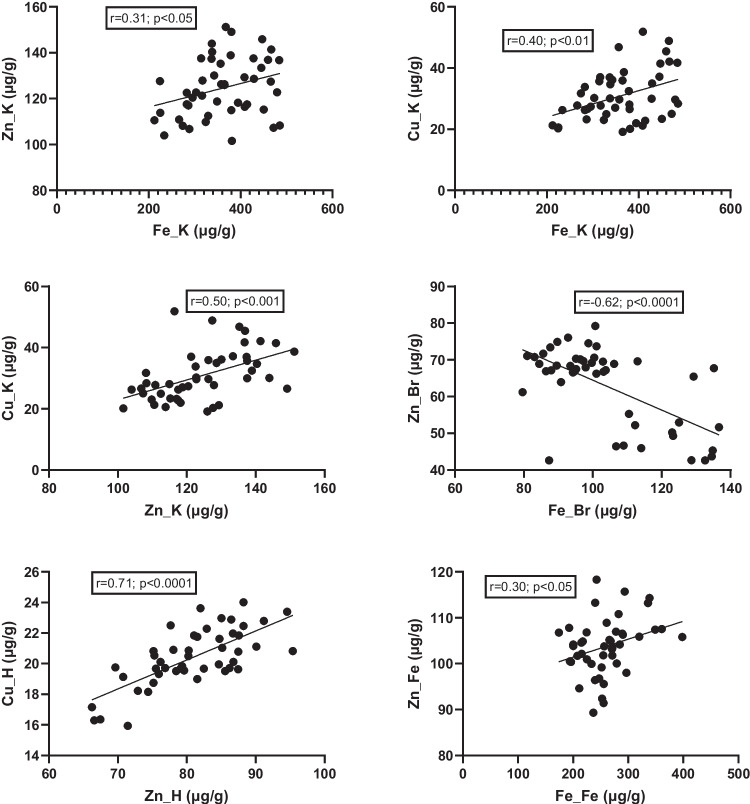
Correlation between minerals in tissues. K, kidney; H, heart; Br, brain; Fe, fetus

**Fig. 13:**
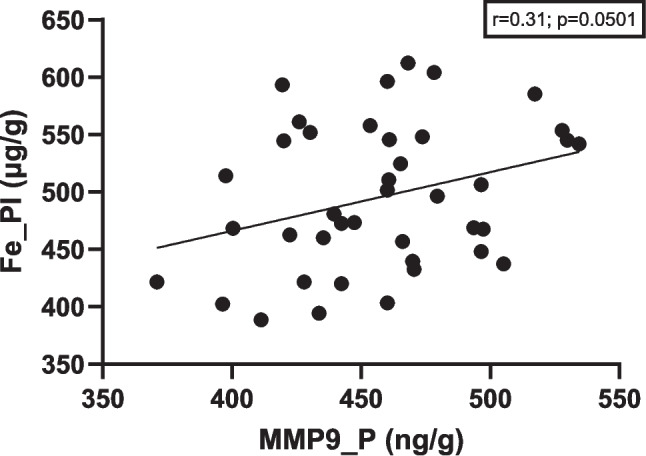
Correlation between iron and MMP-9 in placenta. Pl, placenta; MMP9, metalloproteinase 9

Correlations between parameters were also evaluated. In the kidney, a positive relationship was found between iron, zinc, and copper levels (Fig. 12). Positive correlations were also observed in the heart between zinc and copper levels and in the fetus between zinc and iron content. In contrast, an inverse correlation was noted between zinc and iron levels in the brain. In the placenta, a positive correlation was observed between iron and MMP-9 content (Fig. 13). It is noteworthy that the significance level for this correlation was *p* = 0.0501—borderline for statistical significance—which is why it was still reported.

**Fig. 14:**
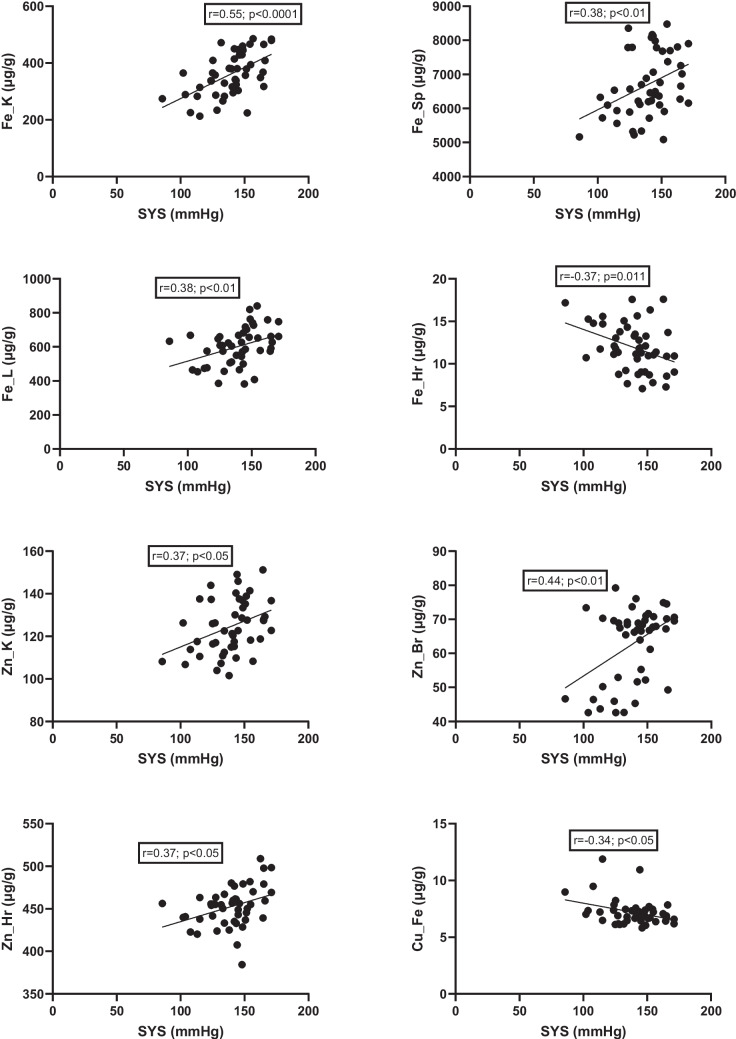
Correlation between SYS and mineral levels in tissues. L, liver; K, kidney; Sp, spleen; Br, brain; Hr, hair; Fe, fetus; SYS, systolic blood pressure

**Fig. 15:**
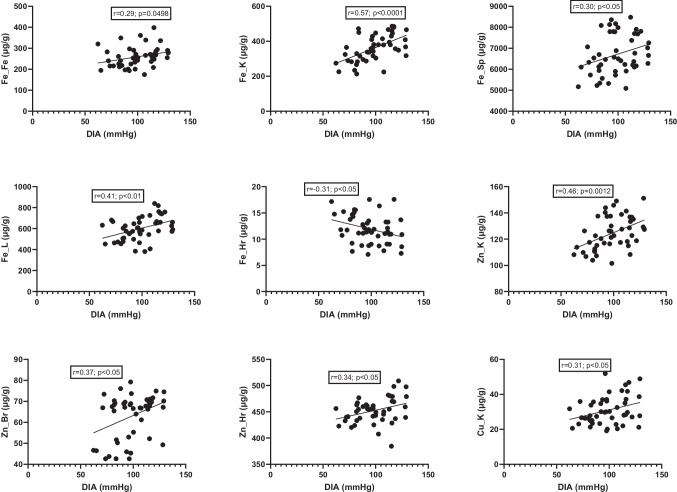
Correlation between DIA and mineral levels in tissues. L, liver; K, kidney; Sp, spleen; Br, brain; Hr, hair; Fe, fetus; DIA, diastolic blood pressure

The study further analyzed relationships between elemental concentrations and blood pressure values (Figs. 14 and 15). A positive correlation was observed between SYS and iron levels in the kidney, spleen, and liver, as well as zinc levels in the kidney, brain, and hair (Fig. 14). In contrast, iron levels in hair and copper levels in the fetus were inversely correlated with SYS (Fig. 14). Diastolic blood pressure was positively correlated with iron levels in the fetus, kidney, spleen, and liver; zinc levels in the kidney, brain, and hair; and copper levels in the kidney (Fig. 15). An inverse correlation was observed between hair iron content and diastolic blood pressure (Fig. 15).

**Fig. 16:**
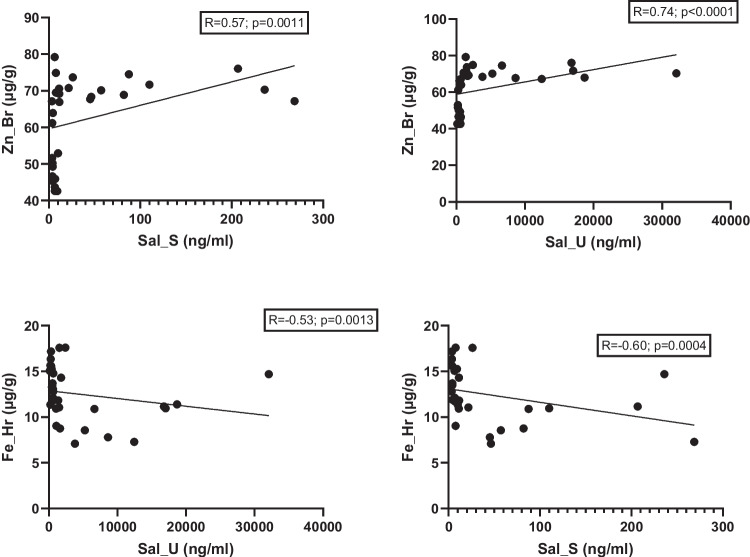
Correlation between salicylate levels and mineral content in tissues. Hr, hair; Br, brain; Sal, salicylates; S, serum; U, urine

The relationship between salicylate levels and trace elements content in tissues was also analyzed. A positive correlation was found between zinc levels in the brain and salicylate concentrations in both serum and urine (Fig. 16). Conversely, a negative correlation was observed between iron levels in the hair and salicylate concentrations in serum and urine (Fig. 16).

## Discussion

This study comprehensively evaluated iron, zinc, and copper status in rats with L-NAME-induced preeclampsia for the first time. Trace elements were analyzed in both maternal and fetal tissues. A scientific novelty of this work lies in the investigation of the effects of dietary salicylates on iron, zinc, and copper status in preeclamptic rats.

A novel finding of this study is the decrease in serum hepcidin levels in rats with L-NAME-induced preeclampsia. Brunacci et al. [32] reported significantly lower hepcidin levels associated with increased serum iron in preeclamptic women. They suggested that increased iron availability from diet and supplementation in preeclamptic women may be related to the inflammatory state characteristic of the condition [32]. However, in the present study, the reduction in maternal hepcidin concentration in the CP group did not result in significant changes in iron content, and the administration of salicylates reversed this L-NAME–induced effect.

Previous studies have shown that nitric oxide (NO) plays a role in iron homeostasis in the placenta during pregnancy and that iron transfer to the fetus is inversely correlated with maternal hepcidin levels [33]. Reduced NO levels in rats receiving an NO synthase inhibitor may lead to dysregulation of placental and fetal iron homeostasis, decreased hepcidin, and increased iron transfer to the developing fetus [33, 34]. Salicylates appear to regulate hepcidin levels, likely by contributing to increased NO synthesis and alleviating symptoms of preeclampsia [35].

The animal model used in this study does not fully replicate the trace element changes typically observed in women with preeclampsia, such as increased iron and copper levels and decreased zinc levels in the blood and placenta [36, 37]. Nevertheless, the observed reduction in hepcidin may represent a key mechanism linking preeclampsia development with iron metabolism. In this study, low hepcidin levels in the CP group were associated with slightly elevated iron concentrations in the fetus, spleen, and bone, as well as slightly increased ferritin levels in the placenta. Moreover, we observed significant positive correlations between iron, zinc, and copper levels in tissues and blood pressure in rats.

It should also be noted that the discrepancies between the results observed in animal models and studies conducted in pregnant women result from the fact that most changes in the parameters are observed depending on the stage of pregnancy in which preeclampsia develops (development of preeclampsia in early or late pregnancy), and in addition, in this model, we have a constant level of trace elements components in the diet (there is no deficit or excess) [38].

The observed correlation between iron concentration in the placenta and MMP-9 levels may indicate that increased iron is associated with placental inflammation [33, 34]. Kaomongkolgit et al. demonstrated that iron can upregulate MMP-9 expression in cells [39]. Based on these associations, the results of our study may suggest a role for the hepcidin–iron–MMP-9 axis in placental dysfunction and the development of preeclampsia.

An important finding of this study is that salicylate intake significantly increased zinc concentrations in the brain and decreased iron levels in the hair. These trends were confirmed by strong correlations between salicylate concentrations in serum and urine and corresponding changes in brain zinc and hair iron levels. Moreover, a high dietary dose of salicylates also increases zinc concentration in the placenta.

Regarding salicylate–iron interactions, prior studies have shown that many aspirin metabolites, including salicylic acid, possess iron-chelating properties [22]. In our study, the reduced iron concentration in the body of pregnant rats may have been offset by minor iron loss through hair, thereby preserving iron levels in other tissues. The significant changes in zinc levels observed in salicylate-treated groups may also be linked to zinc–iron interactions [40, 41]. These interactions are evident in the observed interelement correlations. The decline in iron concentrations following salicylate intake was associated with a nonsignificant decrease in iron in the brain and placenta, possibly prompting a compensatory shift of zinc into these tissues [41].

Additionally, during preeclampsia, oxidative stress and inflammation are elevated in both the placenta and maternal brain. Zinc may be redistributed to these regions to counteract disease effects [42, 43]. In women with preeclampsia, evidence of neuroinflammation and blood–brain barrier disruption has been reported, and zinc has been shown to protect the brain against oxidative stress by contributing to antioxidant activity and membrane stabilization under such conditions [42, 43].

It should also be emphasized that the changes observed following salicylate administration may reflect the limited effectiveness of this intervention in the specific preeclampsia model employed in this study.

## Strengths and Limitations

This study has several strengths as well as limitations. A key strength is the evaluation of the effects of increased dietary salicylate intake on iron, zinc, and copper status in preeclamptic rats. We analyzed element concentrations in tissues such as the placenta and fetus, as well as biochemical parameters related to their metabolism. Ground-based comparative analyses and correlation assessments were also performed.

The main limitation affecting interpretation is the lack of efficacy of the applied therapy and the dosing used. We did not analyze the trace elements in serum due to limited biological material available from rats. Moreover, we did not assess other parameters related to inflammation and oxidative stress, which limits the depth of discussion.

## Conclusion

L-NAME–induced preeclampsia decreases maternal serum hepcidin concentrations, and salicylates abolish this effect. Increased salicylate intake modulates iron and zinc metabolism in preeclamptic rats. Our findings suggest a potential mechanism linking preeclampsia development with iron metabolism. Moreover, the results indicate that salicylate-based prophylactic treatment for preeclampsia may necessitate monitoring of trace element status in pregnant women. To validate these findings, further studies in rat models of preeclampsia are required.

The practical conclusion from this study is that dietary salicylates and aspirin have a similar effect on trace element status in an organism. Dietary salicylates may improve iron metabolism in preeclampsia, but clinical trials are necessary to confirm this effect.

## Data Availability

No datasets were generated or analysed during the current study.
